# Human Fibrinogen: Molecular and Genetic Aspects of Congenital Disorders

**DOI:** 10.3390/ijms19061597

**Published:** 2018-05-29

**Authors:** Giovanni Luca Tiscia, Maurizio Margaglione

**Affiliations:** 1 Atherosclerosis and Thrombosis Unit, I.R.C.C.S. “Casa Sollievo della Sofferenza”, 71013 San Giovanni Rotondo, Italy; g.tiscia@operapadrepio.it; 2 Medical Genetics, Department of Clinical and Experimental Medicine, University of Foggia, 71122 Foggia, Italy

**Keywords:** afibrinogenemia, hypofibrinogenemia, dysfibrinogenemia

## Abstract

Congenital fibrinogen disorders can be quantitative (afibrinogenemia, hypofibrinogenemia) or functional (dysfibrinognemia). To date, several genetic variants have been identified in individuals with fibrinogen disorders. The complexity of the fibrinogen molecules, formed by three non-identical chains and with a trinodal organization, renders the identification of molecular causes and of clinical and biochemical phenotypes very challenging. However, the acknowledgement of the type of molecular defect is crucial for a safer therapy, which is going to improve the clinical management of these patients. In this review, some aspects concerning molecular and clinical findings available on congenital fibrinogen disorders will be discussed.

## 1. Introduction

Fibrinogen is a 45 nm-long plasma glycoprotein with a molecular weight of 340 KDa. It is synthetized in the liver [1] and has a plasma concentration of 1.5–3.5 g/L. Structurally, it consists of 2 identical monomers, each constituted by 3 non-identical polypeptide chains, namely alpha, beta and gamma chains [2,3,4], which interact through 17 crucial inter-chains disulfide bridges, to ensure structural stabilization of the fibrinogen molecules [5,6]. Fibrinogen has a typical trinodal organization, presenting one central (node E) and two distal nodes (nodes D) [7]. The central one is characterized by N-terminal ends of the six polypeptide chains, while the distal ones are made by C-terminal ends of beta and gamma chains, whereas the C-terminal of the alpha chain folds to further form the central node [8,9,10]. The central and distal nodes are connected through coiled-coil regions, built by three α-helices of the three fibrinogen chains, wound around each other.

**Figure 1:**
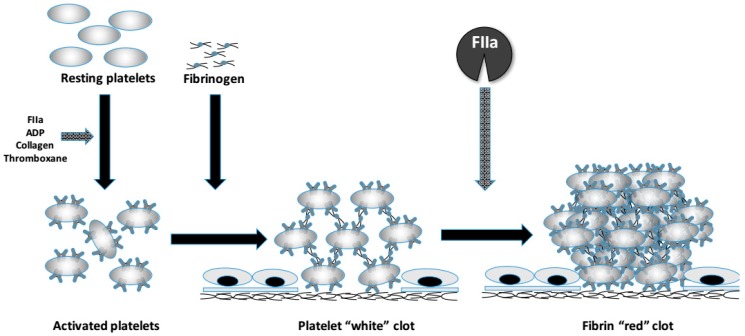
A sketch representing the physiologic role of fibrinogen in the clot formation. The fibrinogen molecule links activated platelets together (white clot) through the alphaIIb–beta3integrin that serves as the fibrinogen receptor. In addition, it is transformed in fibrin by the action of activated thrombin (FIIa), which further stabilizes the fibrin clot by activating FXIII.

The fibrinogen has a well-established role in the clotting enzymatic cascade, representing the fibrin polymer precursor. The impaired mechanism of fibrinogen formation and fibrin polymerization can have a clinical impact on the occurrence and outcome of various diseases, such as coagulopathies, or ischemic stroke and also obstetrical complications [11]. Fibrinogen disorders may also have a clinical impact on afore-mentioned diseases. Fibrinogen is an acute phase protein that is part of the coagulation cascade and is converted into the insoluble protein fibrin during the clotting process. The activated thrombin or Factor II (FIIa) converts fibrinogen to fibrin in response to bleeding. FIIa rapidly proteolyses fibrinogen, releasing two peptides, fibrinopeptide A and fibrinopeptide B, from fibrin and, in turn, forms fibrin monomers, which spontaneously polymerize to form an insoluble gel. Then, fibrin molecules, the major component of the blood clot, combine to form long fibrin threads that entangle platelets, building up a spongy mass that gradually hardens and contracts to form the blood clot, blocking the damaged blood vessel and preventing further bleeding (Figure 1). The polymerized fibrin molecules are held together by non-covalent and electrostatic forces and stabilized by the transamidating enzyme, factor XIIIa, that is produced by the action of FIIa on FXIII. Traditionally, the fibrinogen disorders are categorized as quantitative and functional disorders. For the identification of both classes of disease either immunological assays, which are able to quantify complete absence of circulating fibrinogen molecules, and functional methods, e.g., the Clauss method, a quantitative assay which allows measuring the rate of conversion of clottable fibrinogen into fibrin [12], are used. Fibrinogen disorders may be due to acquired or genetic causes. Liver diseases, cancer, disseminated intravascular coagulation (DIC), post-translational modifications, assay interferences are some of well-known causes of acquired fibrinogen disorders [13]. Inherited disorders of fibrinogen molecules, the topic of this review, are due to genetic alterations occurring within genes coding for the fibrinogen chains. These genes show dissimilar genomic sequences, although they have been found to arise from a common ancestral gene [14]. Recombinant DNA walking experiments allowed to discover that all three genes map on the same chromosomal region, even though they show an opposite transcription orientation [15]. The fibrinogen genes cluster on human chromosome 4, in a region of approximately 50 kb. The gene coding for the fibrinogen alpha chain (gene symbol, *FGA* approved by HUGO Gene Nomenclature Committee) has a 7.6 kb size and consists of 6 exons [16], while the fibrinogen beta chain gene (gene symbol, *FGB*) occupies an 8 kb sized region and presents 8 exons. Finally, the gene coding for the fibrinogen gamma chain (gene symbol, *FGG*) encompasses an 8.5 kb region and presents 10 exons. Congenital fibrinogen disorders are uncommon and can be associated with an altered synthesis, assembly, protein stability and/or with dysfunctional fibrinogen molecules. Afibrinogenemia and hypofibrinogenemia indicate quantitative fibrinogen disorders. They are mostly caused by genetic variations affecting plasma amounts of fibrinogen. On the other hand, dysfibrinogenemia indicates qualitative fibrinogen disorders, which are mostly determined by genetic variations modifying fibrinogen functionality. An extensive review of laboratory issues and an update of genetic diagnosis concerning the fibrinogen disorders has been made by Neerman-Arbez and colleagues [17]. Because congenital fibrinogen disorders are rare, to not duplicate available articles reviewing the literature, we attempt to provide an update, focusing on molecular aspects and genotype-phenotype correlations in quantitative (afibrinogenemia, hypofibrinogenemia) and functional disorders (dysfibrinogenemia). 

## 2. Afibrinogenemia

A diagnosis of afibrinogenemia is made by means of immunological and functional assays, which detect a complete absence of immunoreactive and functional fibrinogen molecules. In 1920, two German physicians described the first case of congenital afibrinogenemia: a 9-year-old boy experiencing bleeding episodes since his childhood [18]. Afibrinogenemia is a rare bleeding disorder, with an estimated prevalence of 1 or 2 to 1,000,000 [19] even if in countries where the consanguinity rate is very high [20], such as in the South India, the number of congenital afibrinogenemia cases increases [21]. In 1999, Neerman-Arbez and colleagues described the first genetic variant associated with afibrinogenemia, a *FGA ~* 11-kb deletion identified in a non-consanguineous Swiss family [22]. The authors showed that the deletion leads to the synthesis of only the first 18 instead of normally produced 625 residues of the fibrinogen alpha chain. Afibrinogenemia is a recessively inherited autosomal trait [23]. While homozygous and compound heterozygous individuals can be clinically symptomatic, heterozygotes usually show no symptoms. The afibrinogenemia can be determined by genetic variants of all three fibrinogen genes, even if variants involving residues composing the alpha chain C-terminal seem to be tolerated, possibly due to the low structural complexity of this fibrinogen domain. Nonetheless, most of genetic variants identified in individuals with afibrinogenemia are found along *FGA* sequences. The vast majority of these variants influences the fibrinogen amount at a DNA-level, producing a ‘null allele’, and in turn, a complete failure of the fibrinogen synthesis. On the other hand, genetic variants have been also described that affect the production of fibrinogen molecules at a protein-level. This class of variants might affect assembly and structural stability of the fibrinogen molecules, which are unable to enter the cell secretory pathway or might influence the fibrinogen secretion. Thus, in individuals with afibrinogenemia, large deletions or frameshift mutations, early nonsense and splice-site variants are usually found [24]. Since it was identified as first genetic cause of the afibrinogenemia, the 11-kb *FGA* deletion was confirmed as a recurrent genetic variant in afibrinogenemic patients [25,26,27]. However, the IVS4 + 1G > T (c.510 + 1G > T, numbered according to the Human Genome Variation Society, HGVS, recommendations) splicing site variant is more common than the 11-kb deletion, especially in individuals of Caucasian ancestry [26,27,28,29,30,31]. In the *FGA* intron 4, a bioinformatics-based study showed that the IVS4 + 1G > T may affect the functionality of the splicing donor site and lead transcriptional machinery to use multiple cryptic splicing sites [32]. As might be expected for a severe phenotype, other ‘null allele’ variants have been reported in afibrinogenemia, although they are less common than the above-mentioned ones. In 2007, Monaldini and colleagues described the last large deletion identified in an afibrinogenemic patient. This deletion involves a 4.1 kb region of *FGA* exon 1 [33]. Other large deletions have been described in afibrinogenemics, involving *FGA* sequences as well [34,35]. In a Japanese patient, who experienced an umbilical vein hemorrhage, Watanabe and colleagues described an 1.2-kb deletion, while in a Thai afibrinogenemic patient showing a complete maternal uniparental disomy, Spena and colleagues described a larger deletion (15-kb deletion). In addition to the IVS4 + 1G > T, further splice site variants have been found, the vast majority with effects on the donor splicing site. However, their effects on splicing process have been experimentally investigated only in a few of them. They would alter the fibrinogen synthesis at a RNA-level, affecting splicing and in turn, as ‘null-allele’ variants, determine the afibrinogenemia. It was demonstrated that these splice site variants, identified in afibrinogenemics, can cause the skipping of a whole exon, the inclusion of intronic nucleotides as new exon sequences, or the use of a cryptic splicing site. Attanasio and colleagues documented the *FGA* exon 3 skipping, due to a 4-bp deletion that includes a donor splicing site [36], while Margaglione and colleagues showed the *FGG* exon 3 skipping, due to the intron 3 G-to-T variant at the +5 nucleotide [37]. Furthermore, Asselta and colleagues described the inclusion of the *FGG* intron 1 in “mature” mRNA, caused by a nucleotide change at the intron +5 position [38]. In a gene expression study performed using HeLa cells, Spena and colleagues showed that two different splicing site variants occurring in *FGB* intron 6 and 7 both cause an aberrant splicing [39]. Furthermore, in *FGG* intron 6, the same research group described the only deep intronic variant (IVS6-320A→T) associated with afibrinogenemia. They experimentally demonstrated that this variant gives rise to a 75-bp pseudo-exon, which includes a premature stop codon [40]. Noteworthy, although *FGA* seems to show the vast majority of large deletions, splicing site variants are quite equally distributed in the three fibrinogen genes. 

As for large deletions, nonsense genetic variants associated with the afibrinogenemia are mostly found in *FGA* sequences. In available fibrinogen databases, more than 16 nonsense variants in *FGA* are reported, whereas only 5 in either *FGB* or *FGG* [41,42]. At the end of 2017, novel nonsense variants in afibrinogenemic patients have been reported. In a 28-year-old afibrinogenemic man presenting with mucocutaneous bleeding and blood losses into muscles, joints, and soft tissues, the Gln180Stop “Martin” variant was identified in *FGB* [43,44]. Naz and colleagues described the novel *FGA* p.Gln183stop and *FGG* p.Lys121stop nonsense variants in two afibrinogenemic patients from Pakistan [45]. Notably, nonsense variants identified in *FGA* sequences were never found in residues belonging to the alpha chain C-terminal end but mainly in exon 5. Conversely, no exons cluster of nonsense variants were observed in other fibrinogen genes. In addition, two nonsense variants in *FGB* (p.Trp467stop, p.Trp470stop)*,* involving amino-acid residues located very close to the C-terminal domain were found [46,47]. These variants give rise to the synthesis of a shorter fibrinogen beta chain, which does not affect the fibrinogen assembly, but it build-up a fibrinogen molecule that fails to be secreted and completely lacks in the bloodstream. Effects on fibrinogen secretion, due to these nonsense variants, contributed to further stress the key role of the beta chain C-terminal end in mechanism leading to fibrinogen secretion. 

It is generally assumed that missense variants disrupt proteins functions and affect protein domains crucial for binding or interaction, as well as impair protein stability. In addition, they may have influences on structural fibrinogen protein stability and assembly or secretion process. As a consequence, fibrinogen molecules firstly fail to pass “cell quality control” and secondly fail to enter the cell secretory pathway. In individuals with afibrinogenemia, while most of “null allele” variants are found within *FGA*, missense variants usually involve amino-acid residues of fibrinogen gamma chain. In a heterozygous compound Italian man with afibrinogenemia, Platè and colleagues described the first missense variant (p.Met70Arg) and demonstrated an impairment of the assembly of fibrinogen molecules [48]. Then, in another heterozygous compound afibrinogenemic, who suffered from intra-articular bleeding, the *FGA* p.Met1Leu missense variant, involving the first amino-acid residue of the fibrinogen alpha chain [49]. A cluster of missense variants was observed in *FGB*. These variants mostly occur within the *FGB* exon 8, which encodes for the C-terminal globular domain, a region that plays a pivotal role in the fibrinogen assembly and secretion [47,50]. 

Finally, frameshift variants, most of them within *FGA,* have been also identified in individuals with afibrinogenemia. These variants can be due to single-base deletions or insertions, or deletion/insertion.

## 3. Hypofibrinogenemia

The diagnosis of hypofibrinogenemia is based on the detection of a proportional reduction of both antigen and functional plasma fibrinogen concentration. Hypofibrinogenemics are usually identified during routine examinations, because they are mostly asymptomatic, and carry heterozygosity of causative variants. In rare bleeding disorder inherited as an autosomal recessive trait, heterozygotes are often asymptomatic. Therefore, individuals with diagnosis of hypofibrinogenemia are thought as heterozygote carriers of afibrinogenemia. Indeed, individuals with laboratory findings suggesting the diagnosis of hypofibrinogenemia are typically investigated as afibrinogenemics, with a first examination of *FGA* sequences and subsequently, of *FGB* exon 8. In hypofibrinogenemics, thrombotic phenotypes associated with fibrinogen beta chain variants have been also described. In a non-thrombophilic woman, who experienced thrombosis events and miscarriages, Casini and colleagues identified the fibrinogen beta chain p.Gly472Val missense change [51]. Furthermore, in a 62-year-old hypofibrinogenemic man with a history of venous thromboses, the fibrinogen beta chain p.Tyr368His variant was found [43]. Protein modeling represents a helpful tool to understand functionality and causality of genetic variants identified in fibrinogen beta chain [52]. A different clinical setting of hypofibrinogenemics presents with distinctive pathological features. This type of patients displays fibrinogen aggregates in endoplasmic reticulum of hepatocytes and mostly of them carry *FGG* variants. It is worth noting that all these gene variants involve residues belonging to the C-terminal end of the fibrinogen gamma chain. In addition, there are individuals with low levels of dysfunctional circulating fibrinogen levels. These individuals are diagnosed as hypodysfibrinogenemics. However, it should be noted that some hypodysfibrinogenemics could be misdiagnosed hypofibrinogenemics, because it can be difficult to correctly figure out fibrinogen assays performed. The vast majority of gene variants identified in hypo-dysfibrinogenemia disorders are missense variants, even if other types of variants have been found, and occur within the C-terminal end of fibrinogen gamma and alpha chains [53]. In addition to heterozygous, hypo-dysfibrinogenemics may carry homozygous or compound heterozygous variants. In this case, it can be difficult to understand possible underlying biochemical mechanisms and, in turn, challenging to predict the clinical phenotype. Indeed, the intermediate phenotype could be the result of poorly assembled and secreted abnormal fibrinogen chains or the combination of two variants, one quantitatively affecting the fibrinogen molecule and the other functionally. 

## 4. Dysfibrinogenemia

Dysfibrinogenemia is the term used to define fibrinogen functional disorders. The diagnosis is suggested by the combined presence of normal antigen amounts and reduced functional levels of circulating fibrinogen. The dysfibrinogenemia is an autosomal dominant trait. At variance with afibrinogenemics, dysfibrinogenemics are mostly found as heterozygous carriers of missense variants, although they can rarely show other type of genetic changes. First description of a dysfibrinogenemic case was provided in 1958 [54], while 11 years after, in a 17-years-old female showing normal quantitative fibrinogen and a non-functional fibrinogen, Blomback and colleagues identified the first molecular cause underlying this clotting disorder—“fibrinogen Detroit” [55]. The young patient showed excessive bleeding during menstruation and was found to carry an arginine-19 to serine change (according to HGVS, p.Arg38Ser) in the fibrinogen alpha chain. However, small deletions [56,57,58,59,60,61], insertions [62,63] in all three fibrinogen genes, as well as splicing variants [64], have been also found. Recently, the frameshift (p.Gly323GlufsX79) variant, ‘Mahdia’, has been identified [65]. Notably, almost 85% of all variants detected are due to nucleotide alterations occurring within the *FGA* exon 2 or *FGG* exon 8, very close to the fibrinogen alpha chain N-terminal end and to fibrinogen gamma chain C-terminal end, respectively [66]. Missense variants identified in dysfibrinogenemics can affect fibrinogen functions at different levels, fibrin polymerization, fibrinopeptide cleavage and/or interactions either at the D-D interface or between the outer D regions and the E central node. Furthermore, missense variants may also alter fibrinolysis process. Although it is suggested to preliminary explore the *FGA* exon 2 or *FGG* exon 8, it is also advised to search for variants involving the fibrinogen gamma chain Arg35 residue, which is frequently found mutated in dysfibrinogenemics [67]. Noteworthy, the Arg35 residue, which represents the cleavage site of thrombin in the fibrinogen alpha chain, may change in cysteine or histidine. Thrombin cleaves the fibrinogen alpha chain and determines the fibrinopeptide A release. The mutated allele may have relevant effects on the polymerization process [25,68], platelet aggregation [69], and fibrinolysis resistance [70]. An additional “hot spot” site is the Arg301 residue, encoded by the *FGG* exon 8 and located in the C-terminal end of the fibrinogen gamma chain. This residue mostly changes to cysteine, more rarely to histidine. Substitution at the Arg301 residue may have functional effect on end-to-end junctions productions, resulting in an abnormal fibrin polymerization process [71]. Other missense variants have been described in dysfibrinogenemics (Human Fibrinogen Database, http://site.geht.org/base-fibrinogene; as accessed 2 April 2018). Although people presenting with a defective fibrinogen may be mostly thought to bleed, dysfibrinogenemics can also suffer from arterial and vein thrombosis, as well as miscarriages. Actually, it has been estimated that approximately 10% of them show thrombosis. In dysfibrinogenemics with thrombosis, variants have been identified in all three fibrinogen genes, and most of them lead to a novel cysteine residue, especially those involving residue changes within the alpha and beta chains. 

## 5. Genotype-Phenotype Correlation in Fibrinogen Disorders

**Figure 2:**
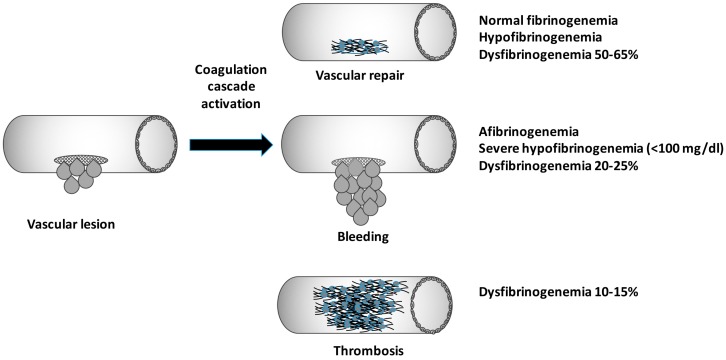
Graphic schematic representation of possible clinical phenotype following a genetic impairment of the fibrinogen molecule.

Correlating clinical symptoms with genotype data is often challenging for physicians, who deal with congenital fibrinogen disorders (Figure 2). Hypofibrinogenemia is associated with fewer bleeding episodes and may not be diagnosed until a traumatic or surgical challenge occurs. In addition, it should be taken into account that mutations causative of fibrinogen aggregates and liver dysfunctions exist. In an Italian proposita showing cytoplasmic inclusions, the p.Gly284Arg was the first variant associated with hypofibrinogenemia and liver accumulation of fibrinogen aggregates [72]. Since then, a series of variants have been associated with fibrinogen storage disease (FSD). The p.His340Asp identified in a young female with fibrinogen hepatic storage and hypertransaminasemia is the last variant reported [73].

The afibrinogenemia is thought as a severe disease and diagnosed individuals can experience life-threatening bleeding. In severe symptomatic patients the prophylactic replacement therapy with fibrinogen concentrate or cryoprecipitate reduce the occurrence of breakthrough life-threatening bleeding (e.g., intracranial bleeding) with a target fibrinogen level of 0.5 g/L [74,75]. However, afibrinogenemics could show also mild symptoms. Thus, prediction of clinical manifestations based on genotype data may be difficult. In congenital fibrinogen disorders, concepts of penetrance and expressivity have a wide room, in attempting to explain the high phenotype variability observed among affected individuals, especially among afibrinogenemics, who could exhibit dissimilar clinical phenotypes, e.g., mild vs. severe hemorrhages, although carrying the same genotype. Afibrinogenemia is often diagnosed in the newborn period because of umbilical cord bleeding and is usually associated with mild-to-severe bleeding. Pregnancy complications, such as miscarriage and placental abruption with fetal loss or premature delivery can occur in women with fibrinogen disorders [76]. In afibrinogenemia, the replacement therapy should be instituted as soon as possible in pregnancy in order to prevent hemorrhagic complications and fetal loss. However, symptoms may vary making phenotype prediction more complex, and in turn, management of pregnancy very difficult, with a patient-by-patient approach [77,78].

Dysfibrinogenemic patients have an unpredictable clinical phenotype, ranging from bleeding to thrombosis, or both; however, most individuals are asymptomatic. In the clinical context of dysfibrinogenemia, the genotype-phenotype correlation would be of help, because a series of gene variants mostly associated with bleeding symptoms (e.g., p.Arg38Ser variant) have been described, while others have been shown to confer a thrombotic phenotype. The fibrinogen alpha chain p.Ser551Cys variant—fibrinogen Caracas V—was observed in a Venezuelan kindred, which showed thrombosis events, probably due to an impaired fibrinolysis [79]. The fibrinogen alpha chain p.Arg573Cys—fibrinogen Dusart/Paris V—was described in a dysfibrinogenemic family presenting with both pulmonary embolism and recurrent thrombosis [80]. The fibrinogen beta chain p.Arg44Cys variant was identified in a dysfibrinogenemic with thrombosis [81]. Other studies confirmed the association between the p.Arg44Cys variant and a dysfibrinogenemia-related thrombosis tendency (see the Human Fibrinogen Database). Finally, the fibrinogen beta chain p.Arg74Cys variant has been found associated with dysfibrinogenemia and venous thrombosis [82]. 

At variance with other rare bleeding disorders, quantitative or functional fibrinogen defects seem to be prone to arterial and venous thrombosis. In this regard, the genotype/phenotype correlation in congenital fibrinogen disorders is difficult due to still unknown mechanisms underlying thrombosis events either in afibrinogenemia or dysfibrinogenemia. Possible hypotheses have been suggested to attempt to explain the occurrence of thrombotic events in fibrinogen disorders. It seems that quantitative deficiency of fibrinogen would lead to an increase in thrombin activity, conferring the thrombotic risk [83,84]. In dysfibrinogenemia, the thrombotic event may be hypothetically caused by a lacking of thrombin binding on defective fibrinogen molecules or by an impaired fibrinolysis. However, it still remains not fully understood how knowledge of the molecular defect may be translated in a clinical event prediction in individuals with fibrinogen disorders. Family and personal history should be taken into account, as well as lifestyle, demographic profile, co-morbidities, and fibrinogen replacement therapy, to attempt to arrange a genotype/phenotype correlation in fibrinogen disorders [85]. It has been hypothesized that variants in different genes, which are well-established to enhance the risk for thrombosis, e.g., factor V Leiden, could actually modulate the risk for thrombosis or the bleeding phenotype in individuals with congenital fibrinogen disorders. However, it is conceivable that unknown variants in other gene loci may play a significant role. Overall, the type of hemostatic challenges, a positive personal history, especially if an arterial thrombosis occurred, age of the propositus at the event, evidences of thrombotic events in relatives, and the knowledge of previous patients carrying the same mutation are key elements for the prediction of clinical manifestations. 

## 6. Conclusions

Congenital fibrinogen disorders are caused by genetic variants occurring within all three fibrinogen genes. However, each gene (*FGA*, *FGB*, *FGG*) differently shows specific variants. Indeed, “nonsense” variants are mostly found within *FGA*, while *FGG* variants are prevalently responsible for missense changes. During the past years, knowledge of underlying molecular genetic bases of fibrinogen disorders has greatly improved. However, in congenital fibrinogen disorders, it still remains to identify potential factors or conditions, which could contribute to the high variability observed in term of clinical phenotypes, ranging from severe bleeding episodes, to thromboembolic events and pregnancy complications. A better standardization of laboratory tests used to diagnose quantitative and functional fibrinogen disorders it is expected, in order to make phenotype identification more precise and reliable. In individuals with fibrinogen defects, future researches should improve diagnostic tools, as global hemostatic tests, such as those based on thromboelastography or the thrombin generation assay, to obtain more comprehensive information on hemostatic tendency and accurately predict the clinical phenotype.
